# Model-based estimation of the impact on rotavirus disease of RV3-BB vaccine administered in a neonatal or infant schedule

**DOI:** 10.1080/21645515.2022.2139097

**Published:** 2022-11-21

**Authors:** Nicholas Geard, Richard Bradhurst, Nefel Tellioglu, Vicka Oktaria, Jodie McVernon, Amanda Handley, Julie E. Bines

**Affiliations:** a School of Computing and Information Systems, The University of Melbourne, Parkville, Australia; b Centre of Excellence for Biosecurity Risk Analysis, School of BioSciences, The University of Melbourne, Parkville, Australia; c Department of Biostatistics, Epidemiology, and Population Health, Faculty of Medicine, Public Health and Nursing, Universitas Gadjah Mada, Yogyakarta, Indonesia; d Center for Child Health – Pediatric Research Office, Faculty of Medicine, Public Health and Nursing, Universitas Gadjah Mada, Yogyakarta, Indonesia; e Department of Infectious Diseases and Victorian Infectious Diseases Reference Laboratory Epidemiology Unit, The Peter Doherty Institute for Infection and Immunity, The Royal Melbourne Hospital and The University of Melbourne, Parkville, Australia; f Melbourne School of Population and Global Health, The University of Melbourne, Parkville, Australia; g Murdoch Children’s Research Institute, Parkville, Australia; h Medicines Development for Global Health, Southbank, Australia; i Department of Gastroenterology and Clinical Nutrition, Royal Children’s Hospital, Parkville, Australia; j Department of Paediatrics, The University of Melbourne, Parkville, Australia

**Keywords:** Rotavirus, RV3-BB vaccine, neonatal schedule, Indonesia, individual-based model

## Abstract

Rotavirus infection is a common cause of severe diarrheal disease and a major cause of deaths and hospitalizations among young children. Incidence of rotavirus has declined globally with increasing vaccine coverage. However, it remains a significant cause of morbidity and mortality in low-income countries where vaccine access is limited and efficacy is lower. The oral human neonatal vaccine RV3-BB can be safely administered earlier than other vaccines, and recent trials in Indonesia have demonstrated high efficacy. In this study, we use a stochastic individual-based model of rotavirus transmission and disease to estimate the anticipated population-level impact of RV3-BB following delivery according to either an infant (2, 4, 6 months) and neonatal (0, 2, 4 months) schedule. Using our model, which incorporated an age- and household-structured population and estimates of vaccine efficacy derived from trial data, we found both delivery schedules to be effective at reducing infection and disease. We estimated 95–96% reductions in infection and disease in children under 12 months of age when vaccine coverage is 85%. We also estimate high levels of indirect protection from vaccination, including 78% reductions in infection in adults over 17 years of age. Even for lower vaccine coverage of 55%, we estimate reductions of 84% in infection and disease in children under 12 months of age. While open questions remain about the drivers of observed lower efficacy in low-income settings, our model suggests RV3-BB could be effective at reducing infection and preventing disease in young infants at the population level.

## Introduction

Rotavirus infection is a major cause of diarrheal deaths and hospitalizations among children younger than 5 years.^1^ While incidence has declined over the last decade, rotavirus gastroenteritis remains a significant cause of morbidity and mortality in children in low-income countries. Most cases of severe rotavirus gastroenteritis occur in children under the age of 2 years, with children under 12 months at highest risk. While 114 countries have introduced rotavirus vaccines into their national immunization program, it is estimated that over 55 million children still lack access to a rotavirus vaccine.^2^ Cost, suboptimal efficacy, and safety concerns remain challenges to the success of rotavirus vaccines.

Four rotavirus vaccines are World Health Organization (WHO) prequalified and available for implementation through GAVI (Global Alliance for Vaccines and Immunization): Rotarix®, Rotateq®, RotaSiil®, and Rotavac®, administered from 6 weeks of age in either a two- or three-dose schedule. These vaccines do not prevent infection but modify the severity of disease. Vaccine efficacy and field effectiveness against severe disease has been found to decline with national income level, from 93% in high-income countries to 51% in low-income countries, with a range of host and environmental factors proposed to contribute to this disparity.^3^

Despite the significant burden of rotavirus disease in Indonesia, rotavirus vaccines are not yet available in the Indonesian National Immunization Program. Key barriers to rotavirus vaccine acceptance in Indonesia include cost, lack of public awareness of rotavirus and its severity, and religious concerns regarding the halal status of the vaccine.^4^ The RV3-BB rotavirus vaccine was developed from the human neonatal rotavirus strain RV3 (G3P6) at MCRI and manufactured under license by PT Bio Farma, Indonesia.^5^ The aim is to provide an affordable, halal manufactured, oral rotavirus vaccine, suitable for introduction into the National Immunization Program in Indonesia. The RV3-BB rotavirus vaccine has been found to be safe and efficacious at reducing the severity of rotavirus gastroenteritis.^6^ Based on a human neonatal rotavirus strain, the RV3-BB rotavirus vaccine provides an opportunity to administer a rotavirus vaccine from birth to provide early protection from severe rotavirus disease and maximize the opportunity to complete a full three-dose schedule.^5–7^ Administration of RV3-BB at birth, 2, and 4 months would align with existing vaccines in the Expanded Program for Immunization (EPI) schedule for Indonesia, including BCG (birth) and DTP-Hib-HepB (2, 3, 4, and 18 months). Mathematical models of rotavirus transmission have been widely used to estimate the direct and indirect benefits of vaccination, typically for the purpose of assessing cost-effectiveness.^8,9^ However, these have uniformly assumed a 2, 4, and 6 month schedules and have not evaluated the potential impact of a neonatal dosing schedule.

The purpose of this study was to estimate the anticipated population-level impact of the RV3-BB vaccine on the prevention of rotavirus gastroenteritis in a low-income setting. We used an individual-based model of rotavirus transmission in an age- and household-structured population, calibrated to match the demography of the Klaten District of Indonesia where the study reported by Bines et al.^5^ was conducted. Using our model, we estimate potential reductions in rotavirus infection and rotavirus gastroenteritis following the introduction of RV3-BB. We compare vaccine impact following delivery according to both an infant schedule with doses at 2, 4, and 6 months, and neonatal schedule with doses at birth, 2, and 4 months.

## Methods

### Demographic model

We modeled a population using a stochastic, spatial, individual-based model, in which individuals are characterized by age and sex. Previous studies of rotavirus transmission have indicated high secondary attack rates in households; we therefore also include household structure in our simulated population. As we are simulating epidemiological dynamics over an extended period to estimate vaccine impact, we also simulate demographic events including the birth, death, and aging of individuals, and the formation and dissolution of households.^10–12^ During a simulation, each person ages and at each (daily) time-step has a probability of leaving home, marrying, having children, or dying. Married couples and those who leave home while unmarried move into newly created households. Each simulated population comprises approximately 150 thousand people occupying around 50 thousand households ranging in size from 1 to 12 people, with a median size of 3 people.

Demographic characteristics of the population, such as age and household size distributions, and fertility and mortality rates, are based on available data on Indonesian demography (https://data.un.org). Households are assigned to a location within a two-dimensional space, with the spatial distribution of households selected to match the population distribution of the Klaten district of Indonesia (https://www.worldpop.org/). See Figures S1 and S2 for indicative age and household size distributions, and Table S1 for parameters governing demographic processes.

### Rotavirus infection and disease model

**Figure 1.**
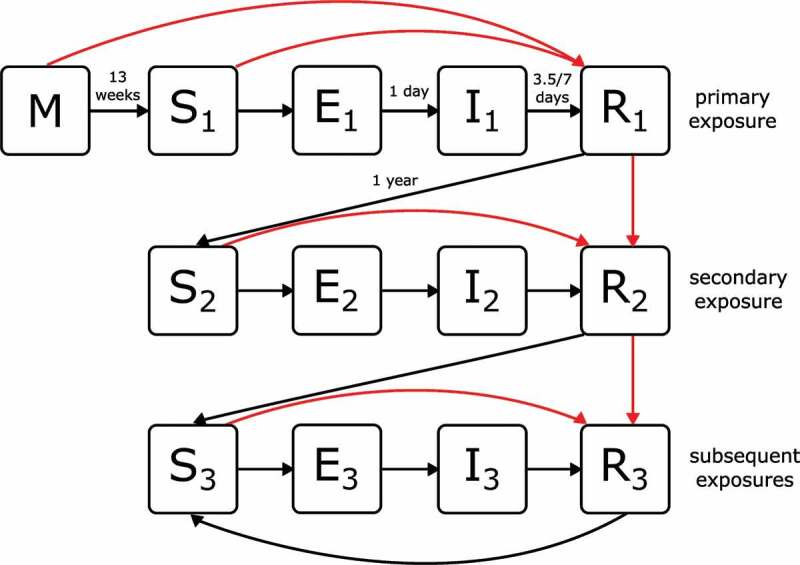
Schematic of the rotavirus infection and disease model to epidemiological data. Infants may be born with maternal immunity (*M*) or susceptible (*S*). Following contact and transmission with an infectious individual, susceptible individuals become exposed (*E*) and then infectious (*I*). Recovered individuals (*R*) are protected from further infection until their immunity wanes, and they once again become susceptible (*S*). Following successive exposures (denoted as subscripts 1, 2 and 3) individuals have different levels of susceptibility, infectiousness, and protection against disease (see Table 1). Black arrows denote transitions occurring due to infection and loss of immunity. Red arrows denote transitions occurring due to vaccination.

We applied a rotavirus-specific transmission model to the population (Figure 1). In addition to an individual’s demographic characteristics, we also keep track of their current state of infection and immunity. Infants born to mothers who have immunity against rotavirus start their life with maternal immunity (*M*). These infants are fully protected against infection until maternal immunity is lost (after a mean duration of 13 weeks), when they become susceptible (*S*). All other infants are susceptible from birth. Upon exposure to infection, susceptible individuals pass through a latent period (*E*) (for a mean duration of 1 day) before becoming infectious to others (*I*). Following recovery (*R*) (after a mean duration of 7 days for severe rotavirus gastroenteritis and 3.5 days for mild rotavirus gastroenteritis or asymptomatic infection), individuals are fully protected against further infection for a mean duration of 1 year, before waning again into a susceptible state (*S*). The model includes parameter values for the duration of maternal immunity, latent and infectious durations, and the duration of immunity following infection (Table 1).

**Table 1.** Fixed parameter values and sources.

Parameter	Value	Reference
Mean duration of maternal immunity (*M*)	13 weeks	Linhares et al.^13^
Relative risk of infection (compared to *S*_1_) following:		
*First infection (S*_2_)	0.62	Velázquez et al.^14^
*Second and subsequent infections (S*_3_)	0.37	Velázquez et al.^14^
Mean duration of latent period (*E*)	1 day	de Blasio et al.^15^
Proportion of infections with any RVGE (severe RVGE):		
*First infection* (*I*_1_)	0.47 (0.13)	Pitzer et al.^16^
*Second infection* (*I*_2_)	0.25 (0.03)	Pitzer et al.^16^
*Third and subsequent infections* (*I*_3_)	0.2 (0.0)	Pitzer et al.^16^
Mean duration of infectiousness:		
*Severe RVGE*	7 days	Ward et al.^17^
*Mild RVGE/asymptomatic*	3.5 days	Wilde et al.^18^
Relative infectiousness (compared to severe RVGE):		
*Mild RVGE*	0.5	Koopman et al.^19^
*Asymptomatic*	0.2	Koopman et al.^19^
Mean duration of full immunity following recovery (*R*)	1 year	Chiba et al.^20^

In line with empirical data and existing models, we assume that the relative risk of acquiring infection decreases for each infection after the first.^8^ In the transmission model, this is represented via three susceptible states, *S*_1_, *S*_2_, and *S*_3_, corresponding to individuals who have experienced zero, one, and two or more infections, respectively (Figure 1).

Infections can be asymptomatic or result in mild or severe rotavirus gastroenteritis. As with susceptibility, we assume that the relative risk of developing rotavirus gastroenteritis declines with successive infections (denoted by states *I*_1_, *I*_2_, and *I*_3_). We further assume that rotavirus infections that are asymptomatic or cause mild disease are of shorter duration and reduced infectiousness compared to those that cause severe disease. The model includes parameters for susceptibility, disease risk, and onward transmission (Table 1).

Recovery from infection is followed by a period of full protection during which reinfection is not possible (denoted by states *R*_1_, *R*_2_, and *R*_3_). All *R* states are assumed to be equivalent in terms of their degree and duration of immunity and are only differentiated to enable tracking of immune maturation. After waning of full protection, an individual becomes susceptible again, with a reduced risk of symptomatic infection as described above.

### Rotavirus transmission model

#### Within-household transmission

The transmission of infection within a household is modeled using a stochastic non-spatial kernel. Household members are assumed to mix homogeneously inside the house, and all susceptible individuals are deemed to be at risk of infection each day that there is an infectious individual living in the household. The daily probability of transmission *P*_*t*_ between an infectious and a susceptible individual is calculated as *P*_*t*_ = *P*_*b*_*W*_*i*_*W*_*s*_*W*_*x*_, where *P*_*b*_ is the baseline daily probability of transmission *P*_*b*_ = 0.0767, that is adjusted according to the infectiousness of the source individual *W*_*i*_, the susceptibility of the at-risk individual *W*_*s*_, and a seasonal influence on transmission *W*_*x*_. This is a model that captures individual variability in the infectiousness and susceptibility of household members, as well as the stochastic nature of transmission.

The baseline probability of transmission within a household was estimated based on a study by Lopman et al.^21^ which reported a 55% chance of transmission over the course of an average 10-day infectious period: *P*_*b*_ = 1 − (1 − 0.55)^(1*/*10)^ = 0.0767. Values for *W*_*i*_ and *W*_*s*_ are shown in Table 1 and were derived from studies reported by Velázquez et al.^14^ and Koopman et al.,^19^ respectively. Transmission probability was increased by 10% in summer and decreased by 10% in winter, based on reported rotavirus incidence in low- and middle-income countries in tropical climates.^22^

#### Between-household transmission

The transmission of infection between households is modeled using a stochastic spatially explicit contact-based spread pathway. Modeling between-household transmission separately to within-household transmission captures the multi-scale nature of an epidemic in that the mechanisms and rates of spread within sub-populations are often quite distinct from those between sub-populations.^23^

The number of daily contacts that an infectious individual has with individuals outside of their household is determined by their age and their disease state (Table 2). People with mild and severe disease have their inter-household contacts reduced by 60% and 95%, respectively, on the assumption that they are less likely to travel beyond their household while unwell. For each contact event, a target individual is chosen from another household based on an age bracket-based contact matrix and distance distribution. The probability that a contact then results in transmission *P*_*t*_ is calculated as described above for within-household contacts, with *P*_*b*_ = 0.15, based on a study reported by Senturia et al.^24^ which reported a 15% chance of transmission from a one-off contact event.

**Table 2.** Contact rates for non-household contacts.

Parameter	Value
Mean daily number of non-household contacts	12 (0–3 years) 14 (4–16 years) 21 (17–49 years) 17 (50–65 years) 10 (65+ years)
Contact suppression weighting	1.0 (subclinical) 0.4 (mild disease) 0.05 (severe disease)

### Rotavirus vaccination model

Rotavirus vaccination is thought to have a comparable effect on immunity to natural infections, conferring some protection against infection and stronger protection against disease.^14,25^ Successive infections with rotavirus induce increased protection, and here we assume that successive successful vaccine doses (i.e., those that produce a detectable response) contribute to immune maturation in the same fashion. In model terms, this corresponds to individuals progressing through recovered states *R*_1_, *R*_2_, and *R*_3_ with each successful vaccine dose. We further assume that individuals who have experienced natural infection prior to receiving a vaccine dose will never revert to an earlier stage of immune maturation. That is, an individual who has been infected (and recovered) prior to receipt of their first vaccine dose would already be in state *R*_1_; therefore, successful seroconversion following their first dose would move them to state *R*_2_.

We use data on cumulative vaccine response (as reported in Figure 3 and Table S3 of Bines et al.^5^) to parameterize the proportion of recipients, by dose and schedule, who are protected by vaccination (Table 3). In that study, cumulative vaccine response was defined as the proportion of study participants displaying either a serum immune response (a serum rotavirus IgA antibody titer or a serum neutralizing antibody titer three times as high as the titer at baseline) 28 days after administration of the vaccine and/or shedding of RV3-BB between days 3 and 7 after the vaccine was received. For example (in the case of the neonatal schedule) 23% of infants receiving dose 1 would have immunity equivalent to a primary infection (*R*1).

**Table 3.** Cumulative vaccine response.

	Infant Schedule	Neonatal Schedule
Dose 1	42%*	23%
Dose 2	87%	53%*
Dose 3	99%	94%

*Values may be underestimated due to absence of sera collection at this timepoint.

Following dose 2, those 23% of infants would have immunity equivalent to a secondary infection, while a further 30% (i.e., 53–23%) would have immunity equivalent to a primary infection. Following dose 3, 23% of infants would have immunity equivalent to a third infection, with 53% (41%) having immunity equivalent to a secondary (primary) infection (*R*2 or *R*1). The proportion of infants in each immunity class following one, two and three doses is shown in Figure S3.

### Model calibration

Demographic and disease model parameters were set as described in Sections 2.1–2.3. To establish that the model produced plausible epidemiological patterns, we compared output on incidence of infection and disease from the baseline scenario, with no vaccination, to values reported in the literature.

There are relatively limited data available on incidence of rotavirus infection and disease in Indonesia. Using data from the Global Burden of Disease Study 2016,^26^ Troeger et al.^1^ report estimates of 8.8 deaths per 100,000 due to rotavirus in children under 5 years, and 538.7 cases of rotavirus per 1000 in children under 5 years (95% CIs: 369.3, 776). Drawing on an earlier systematic review of global studies,^27^ Debellut et al.^28^ report estimates of 78.2 cases of non-severe rotavirus gastroenteritis per 1000 in children under 5 years (95% CIs: 58.3, 104.8) and 21.8 cases of severe rotavirus gastroenteritis per 1000 in children under 5 years (95% CIs: 11.8, 35.3).

Incidence of infection and disease in adults is more difficult to quantify, as most infections in these groups are likely to be asymptomatic or very mild, and little data is available. We therefore focused on rates of infection and disease in children under five in assessing baseline model outputs.

### Simulation of alternate vaccination scenarios

Three scenarios were simulated (with 10 stochastic runs per scenario) to allow the estimation of the likely impact of alternative RV3-BB schedules on rates of infection and disease:

- **Baseline**: No vaccination.
- **Infant**: Vaccination according to the infant schedule (2, 4, and 6 months).
- **Neonatal**: Vaccination according to the neonatal schedule (0, 2, and 4 months).

For all scenarios, we first simulated an initial “burn-in” period of 30 years with no vaccination to establish endemic transmission in the population. This resulted in a baseline population of approximately 153 thousand individuals, of which 90% had an exposure state of R3, 3% an exposure state of R2, 3% an exposure state of R1, and 4% were unexposed.

In all vaccination scenarios, we assumed coverage of 85% across the population. Vaccination status was assumed to be correlated within households. That is, the first child in a household eligible for vaccination had an 85% chance of being vaccinated. Thereafter, all subsequent children born into a household were vaccinated or not based upon the status of the first eligible child. Vaccination was modeled to commence for newborn infants at the target coverage level; there was no ramp-up or catch-up vaccination.

We measured the incidence of any infection, mild disease, and severe disease by month of age for children under 6 months, then 3 months of age for children under 2 years, and then in age ranges (2–3 y, 3–4 y, 4–7 y, 7–11 y, 11–17 y, 17–30 y, 30+ y).

### Sensitivity analysis: vaccine coverage

While nationally reported coverage for DTP-Hib-HepB3 (Diphtheria Tetanus Pertussis-Haemophilus Influenza-Hepatitis B 3 doses) is over 80% in Indonesia, inequalities are observed across provinces and socioeconomic groups.^29^ We investigated the sensitivity of our model results to vaccine coverage by examining lower coverage levels of 55% and 25%.

### Sensitivity analysis: maternal immunity

**Figure 2.**
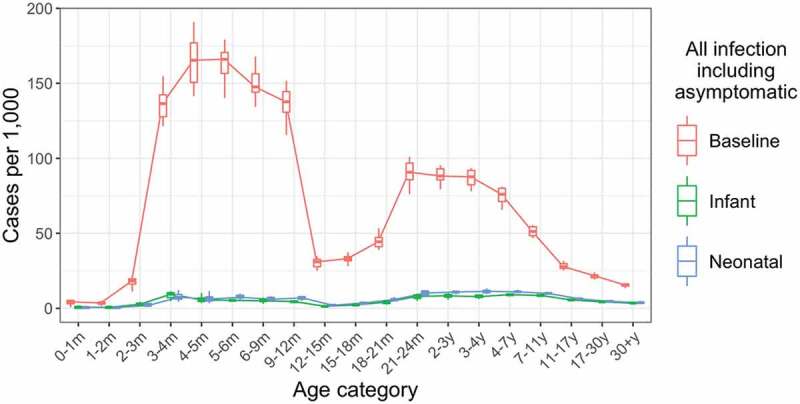
Annual incidence of all rotavirus infections, including asymptomatic infections, by age in the baseline, infant schedule and neonatal schedule scenarios. Incidence values are calculated over a five-year period, five years after the introduction of vaccination. Each boxplot shows the median, IQR and range over 10 simulation runs.

Our baseline assumption was that infants born to a mother with immunity had a 90% chance of acquiring maternal antibodies and that these antibodies provide immunity for, on average, the first 3 months of life (Table 1). This protective effect can be seen in the lower incidence in the first few months of life before maternal immunity wanes and rates of infection increase (Figure 2). We investigated the sensitivity of our model results to assumptions about maternal immunity by examining a scenario in which the probability of acquiring immunity from maternal antibodies was halved to 45%.

## Results

### Incidence of infection and disease by age

Across the 10 baseline scenario simulations, our model produced a median of 84.5 rotavirus infections per 1000 in children under 5 years (95% CIs: 75.4, 90.0), with 28.2 cases of non-severe disease (95% CIs: 24.4, 29.9) and 10.4 cases of severe disease (95% CIs: 9.4, 11.7).

We compared the effect of vaccination with RV3-BB according to an infant (2-, 4-, and 6-month doses) and neonatal (birth-, 2-, and 4-month doses) schedule to the baseline scenario with no vaccination, over a 10-year period. Both vaccine schedules produced substantial reductions in both infection (Figure 2) and disease (Figures S4 and S5).

For infants under 12 months of age, vaccination according to the infant schedule reduced annual incidence of infection by 96% (from 112.9 per 1000 to 4.4 per 1000) and vaccination according to the neonatal schedule reduced annual incidence of infection by 95% (from 112.9 per 1000 to 5.4 per 1000) compared to the baseline scenario with no vaccination.

In this age group, the infant and neonatal schedules reduced annual incidence of any disease by 96% (from 52.9 per 1000 to 2.4 per 1000) and 95% (from 52.9 per 1000 to 2.5 per 1000), respectively, and annual incidence of severe disease by 96% (from 14.8 per 1000 to 0.6 per 1000) and 95% (from 14.8 per 1000 to 0.7 per 1000), respectively.

Vaccination also reduced infections among unvaccinated cohorts, indicating the potential for substantial indirect impact: annual incidence of infection and any disease was reduced by 78% (from 16.7 per 1000 to 3.6 per 1000) and by 78% (from 3.3 per 1000 to 0.73 per 1000), respectively, in adults above 17 years of age for the infant schedule, and by similar amounts for the neonatal schedule.

### Sensitivity analysis: vaccine coverage

**Figure 3.**
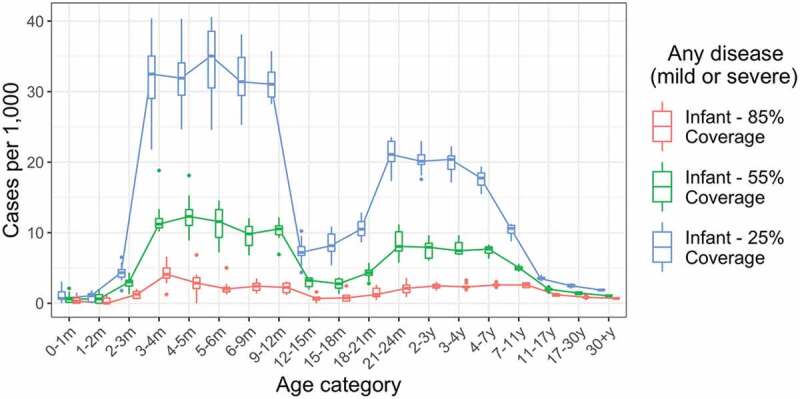
Impact of lower vaccine coverage. Annual incidence of all rotavirus infections, including asymptomatic infections, by age for baseline assumption of 85% vaccine coverage, and alternative moderate (55%) and low (25%) vaccine coverage scenarios. Incidence values are calculated over a five-year period, five years after the introduction of vaccination. Each boxplot shows the median, IQR and range over 10 simulation runs.

We examined the impact of lower vaccine coverage levels of 55% and 25%, compared to our baseline assumption of 85% (Figure 3). At 55% vaccine coverage, annual incidence of infection was reduced by 84% (from 112.9 per 1000 to 18.1 per 1000), annual incidence of any disease was reduced by 84% (from 52.9 per 1000 to 8.3 per 1000), and annual incidence of severe disease was reduced by 84% (from 14.8 per 1000 to 2.4 per 1000), respectively, in infants under 12 months of age. As may be anticipated, vaccine impact drops more markedly when coverage is reduced to 25%, with both direct and indirect protective effects reduced.

### Sensitivity analysis: maternal immunity

**Figure 4.**
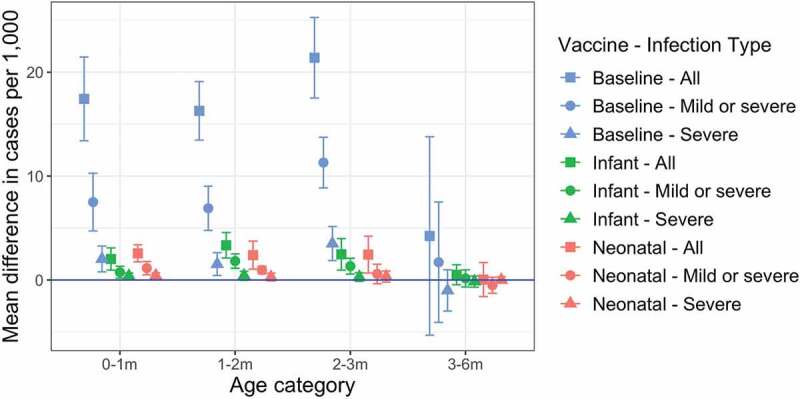
Impact of reduced maternal immunity on annual incidence when the probability of acquiring maternal immunity is halved. Annual incidence of all rotavirus infections, any disease, and severe disease only are shown for the baseline (no vaccination), infant schedule, and neonatal schedule scenarios. Incidence values are calculated over a five-year period, five years after the introduction of vaccination. Each error bar shows the median, 95% CIs over 10 simulation runs.

We examined a scenario in which the probability of acquiring immunity from maternal antibodies was 45%, compared to our baseline assumption of 90% (Figure 4). Compared to our baseline scenario, annual incidence of infection increased by 210% (from 8.7 per 1000 to 27.1 per 1000) over the first 3 months of life.

## Discussion

Rotavirus gastroenteritis remains a significant health burden in many low- and low-middle-income countries despite the development of effective rotavirus vaccines. Model-based estimates of vaccine impact can provide a valuable insight into the potential cost-effectiveness of vaccination and support decision-making for the introduction of rotavirus vaccines into the National Immunization Programs. This study used an individual-based model to estimate the impact of oral human rotavirus vaccine RV3-BB delivered under both an infant and neonatal schedule.

Our model of rotavirus transmission in a low-income setting showed that RV3-BB vaccination could substantially reduce rotavirus infection and disease, particularly in young infants. As modeled here, the infant and neonatal schedules of RV3-BB both produced comparable reductions in infection and disease in young infants, despite observed differences in seroconversion rates.^5^ These reductions in infection and disease following vaccination are a direct consequence of bringing forward the development of an immune response, without the consequence of disease, compared to exposure to natural infection. As the first episode of rotavirus infection is usually the most severe, replacing this exposure event with vaccination is particularly important. RV3-BB vaccine differs from other rotavirus vaccines in two key respects. First, the intrinsic characteristics of the strain that is the basis for the RV3-BB vaccine (G3P3) can infect the newborn gut without causing disease, enabling a lower risk route to immune maturation. Second, the ability to commence delivery of RV3-BB at birth decreases the duration of the high-risk period between birth (and waning of maternal immunity) and direct protection.

This model is the first that we are aware of to make use of published estimates of RV3-BB vaccine efficacy^5^ and the first to make use of a stochastic individual-based model. This individual-based model formulation has several advantages compared to a traditional mathematical model. First, an individual-based model enables a more detailed representation of individual histories of exposure to rotavirus infection and vaccination, thus capturing heterogeneity in immune maturation across a population. Second, the inclusion of household structure captures an important setting for rotavirus transmission, enabling a more accurate representation of the indirect effects of vaccination.^30^ Sensitivity analyses suggest that even if high vaccine coverage levels were not reached, moderate coverage could provide sufficient direct and indirect protection to substantially reduce incidence of infection and disease in young children, as well as reduce incidence across the broader population. While previous rotavirus models have been calibrated based on different vaccine formulations, overall estimates of vaccine impact are broadly similar when vaccine coverage is high. Compared to existing models (e.g., by Van Effeltere et al.^9^), our model suggests that vaccine impact could be maintained to a greater extent as coverage decreased, suggesting that the effects of indirect protection may be stronger in a spatially structured population.

Any modeling study necessarily abstracts reality and hence is subject to limitations. As the primary focus of our model was on the ability of RV3-BB to reduce disease in young children, we did not incorporate long-term waning of protective immunity. Therefore, we did not observe elevated levels of infection and disease among older adults, as has been observed in other populations, and captured in other rotavirus models (e.g., as reported by Pitzer et al.^8^). While we did observe reductions in incidence of infection, consistent with epidemiological studies,^31^ there are insufficient seroprevalence data on adults in our target population to validate this aspect of the model.

Considerable uncertainty remains about the drivers of reduced efficacy of rotavirus vaccines in low-income settings.^32^ One potential explanatory factor is that maternal antibodies interfere with an infant’s primary response to rotavirus vaccines, reducing their efficacy, although a prior study has found that presence of maternal antibodies was not associated with a reduced response to RV3-BB.^33,34^ However, we did demonstrate that indirect protection resulting from infant vaccination reduced incidence across the age spectrum, including adults of childbearing age. It is possible that reduced incidence of infection in mothers could lead to reduced transfer of maternal antibodies to newborns and increased vaccine efficacy. As demonstrated by our sensitivity analysis, effective maternal immunity does protect newborns in their first months of life, consistent with observations.^34^ Any reduction in maternal immunity arising from reduced circulation of rotavirus could strengthen the argument for a neonatal vaccine schedule that initiates the pathway to immune maturation at the earliest possible point in time.

## Conclusion

When making decisions about the introduction of a new vaccine into a National Immunization Program, policy-makers rely on the best available estimates of the direct and indirect impacts of the vaccine as it relates to the target population. This data is not always available, particularly for low- and low-middle-income country settings. Thus, mathematical modeling can help by providing estimates of the likely impact of a new vaccine based on trial data and population characteristics. The findings of this study support the inclusion of a rotavirus vaccine in Indonesia’s Expanded Program for Immunization to reduce the burden of rotavirus disease.

The strength of individual-based models compared to compartmental models is that they can represent rich patterns of heterogeneity present in real populations. Here, we have focused on immune maturation and household structure. Future research could extend the model presented here to explore the impact of interactions between adult infection and immunity and behaviors such as breastfeeding that may alter neonatal protection against rotavirus infection and disease. Furthermore, we have focused primarily on the short-term impacts of a rotavirus vaccination program. Models that include demographic dynamics also offer the ability to explore long-term trends in immunity and demographic change.^35^ In particular, such models could be used to assess potential impacts of immunization programs on rotavirus strain replacement, with implications for ongoing vaccine effectiveness.^36^

## Supplementary Material

Supplemental Figures and Tables

## Data Availability

Deidentified group data may be available for sharing on application via the corresponding author. This application must include the relevant proposal detailing the intended use of the data, the ethics approval for this proposal and requires a signed data sharing agreement. Additional study documents including the study protocol, statistical analysis plan and informed consent are available on application via the corresponding author on publication of this report.
