# Supplementary material for: Model-based estimation of the impact on rotavirus disease of RV3-BB vaccine administered in a neonatal or infant schedule
Source: Hum Vaccin Immunother. 2022 Nov 21;18(6):2139097. doi: 10.1080/21645515.2022.2139097 (PMC9746532; doi:10.1080/21645515.2022.2139097)
Supplement: Supplemental Figures and Tables [file KHVI_A_2139097_SM1816.docx]

Model-based estimation of RV3-BB vaccine impact under infant and neonatal schedules – Supplementary Information

Nicholas Geard^a*^, Richard Bradhurst^b^, Nefel Tellioglu^a^, Vicka Oktaria^c,d^, Jodie McVernon^e,f,g^, Amanda Handley^g,h^, Julie E. Bines^g,i,j^

^a^School of Computing and Information Systems, The University of Melbourne, Parkville, Australia; ^b^Centre of Excellence for Biosecurity Risk Analysis, School of BioSciences, The University of Melbourne, Parkville, Australia; ^c^Department of Biostatistics, Epidemiology, and Population Health, Faculty of Medicine, Public Health and Nursing, Universitas Gadjah Mada, Yogyakarta, Indonesia; ^d^Center for Child Health – Pediatric Research Office, Faculty of Medicine, Public Health and Nursing, Universitas Gadjah Mada, Yogyakarta, Indonesia; ^e^Department of Infectious Diseases and Victorian Infectious Diseases Reference Laboratory Epidemiology Unit, The Peter Doherty Institute for Infection and Immunity, The Royal Melbourne Hospital and The University of Melbourne, Parkville, Australia; ^f^Melbourne School of Population and Global Health, The University of Melbourne, Parkville, Australia; ^g^Murdoch Children’s Research Institute, Parkville, Australia; ^h^Medicines Development for Global Health, Southbank, Australia; ^i^Department of Gastroenterology and Clinical Nutrition, Royal Children's Hospital, Parkville, Australia; ^j^Department of Paediatrics, The University of Melbourne, Parkville, VIC, Australia

^*^Corresponding author: nicholas.geard@unimelb.edu.au

# Supplementary Figures


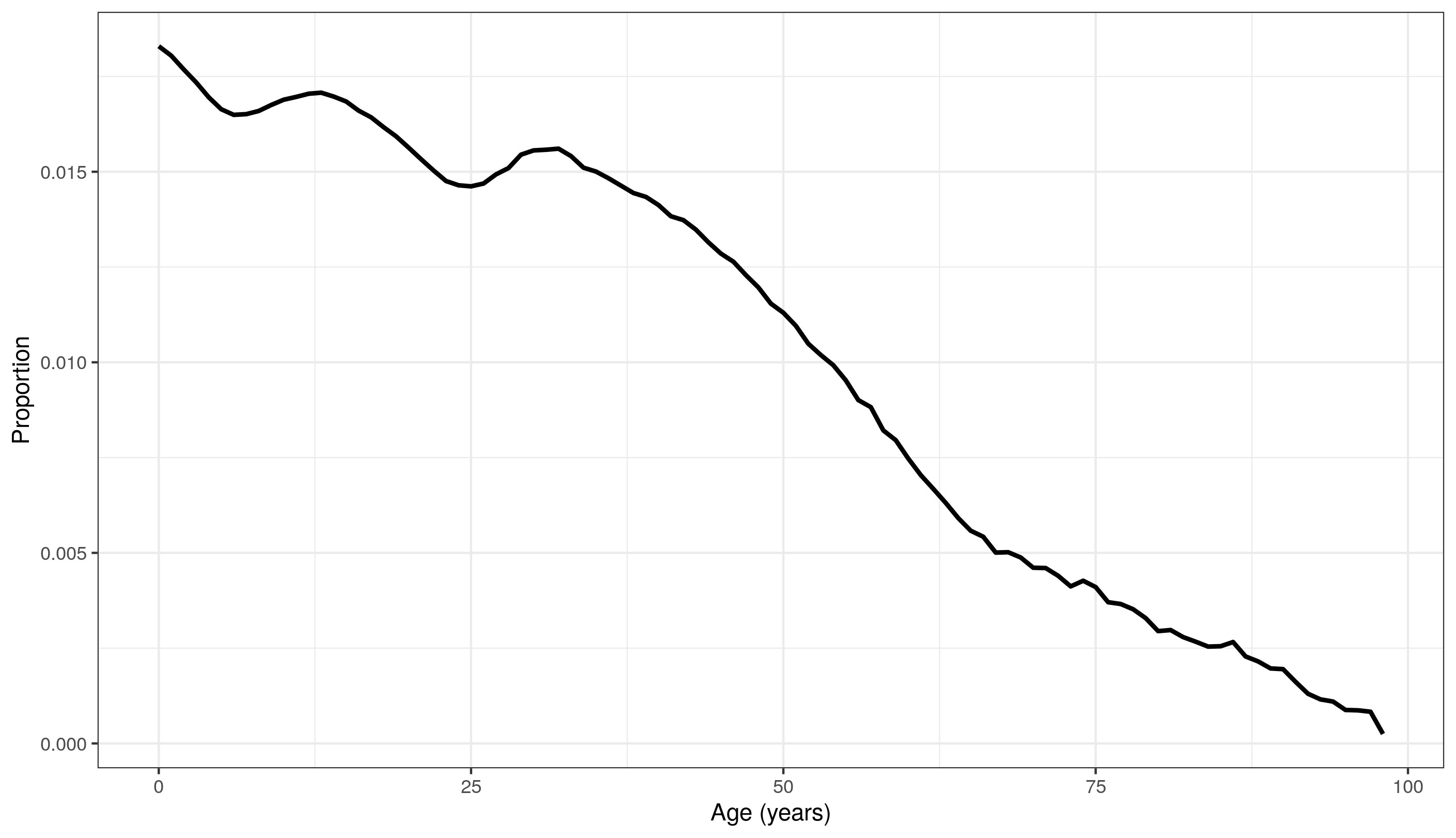


Figure S1: Age distribution of the simulated population.


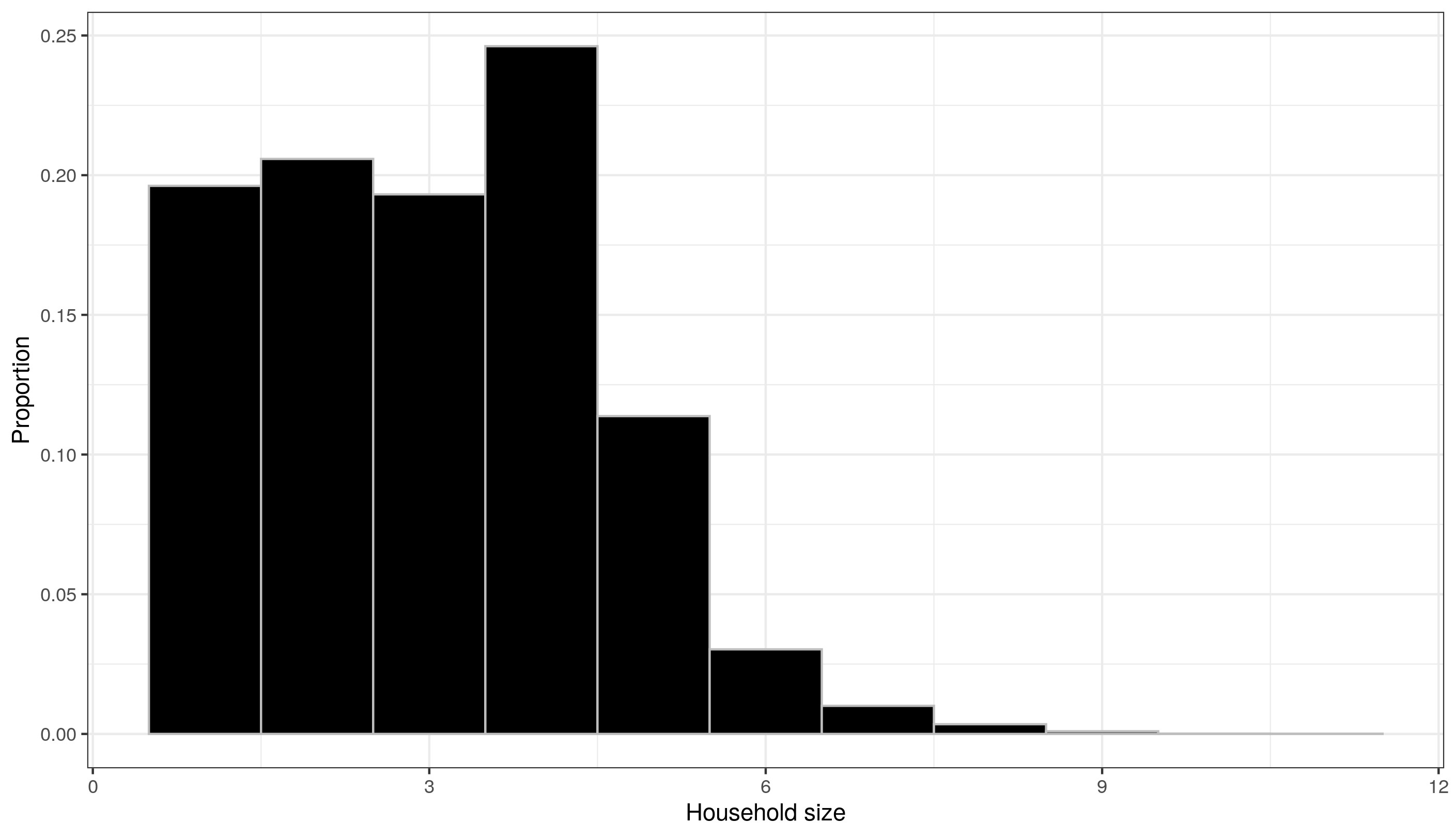


Figure S2: Household size distribution of the simulated population.


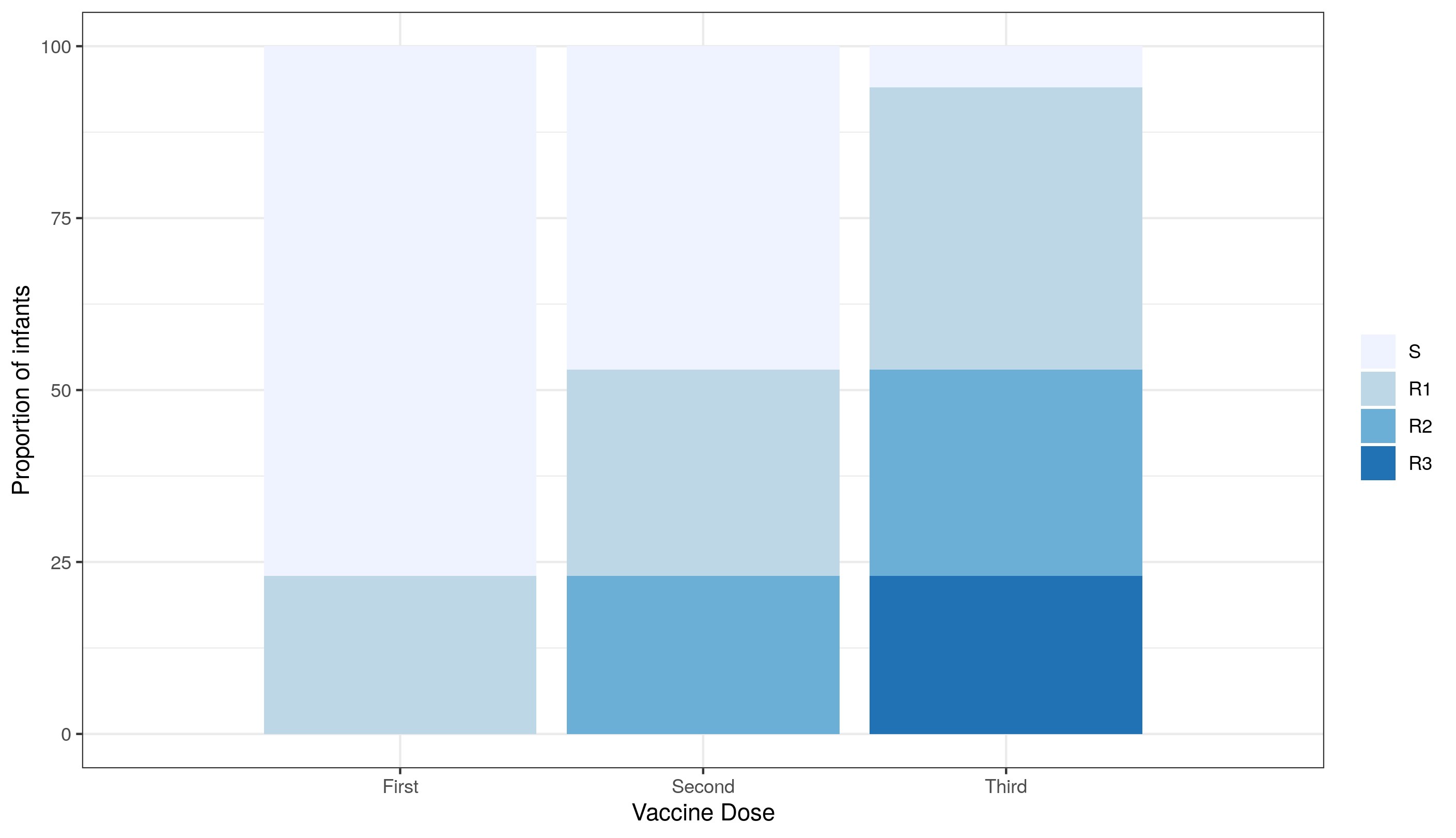


Figure S3: Proportion of infants in each immunity class following receipt of their first, second and third vaccine dose. Susceptible (*S*) infants remain fully susceptible to subsequent infection and disease. Infants who seroconvert following one or more of the vaccine doses (*R*_1_, *R*_2_ and *R*_3_) have reduced susceptibility to infection, reduced transmissibility upon breakthrough infection, and are at reduced risk of developing mild or severe rotavirus gastroenteritis.


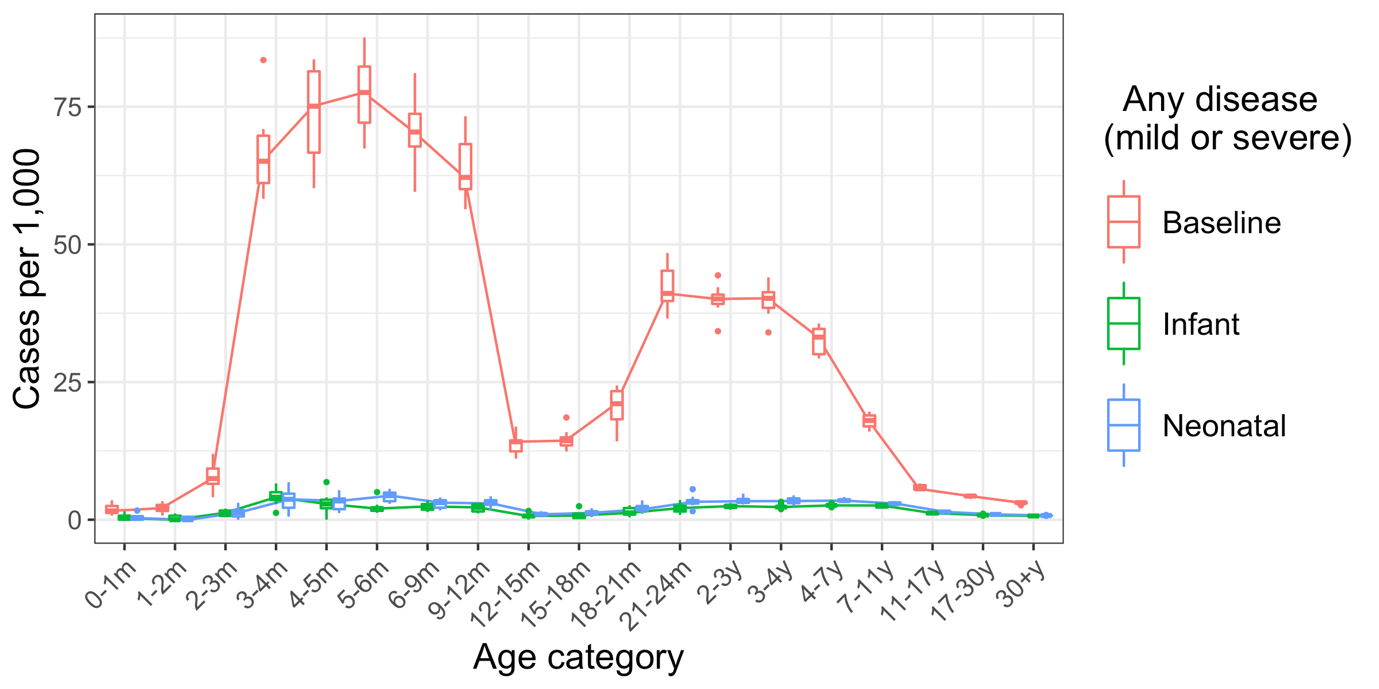


Figure S4: Annual incidence of any rotavirus disease (mild or severe) by age in the baseline, infant schedule and neonatal schedule scenarios. Incidence values are calculated over a five-year period, five years after the introduction of vaccination. Each boxplot shows the median, IQR and range over 10 simulation runs.


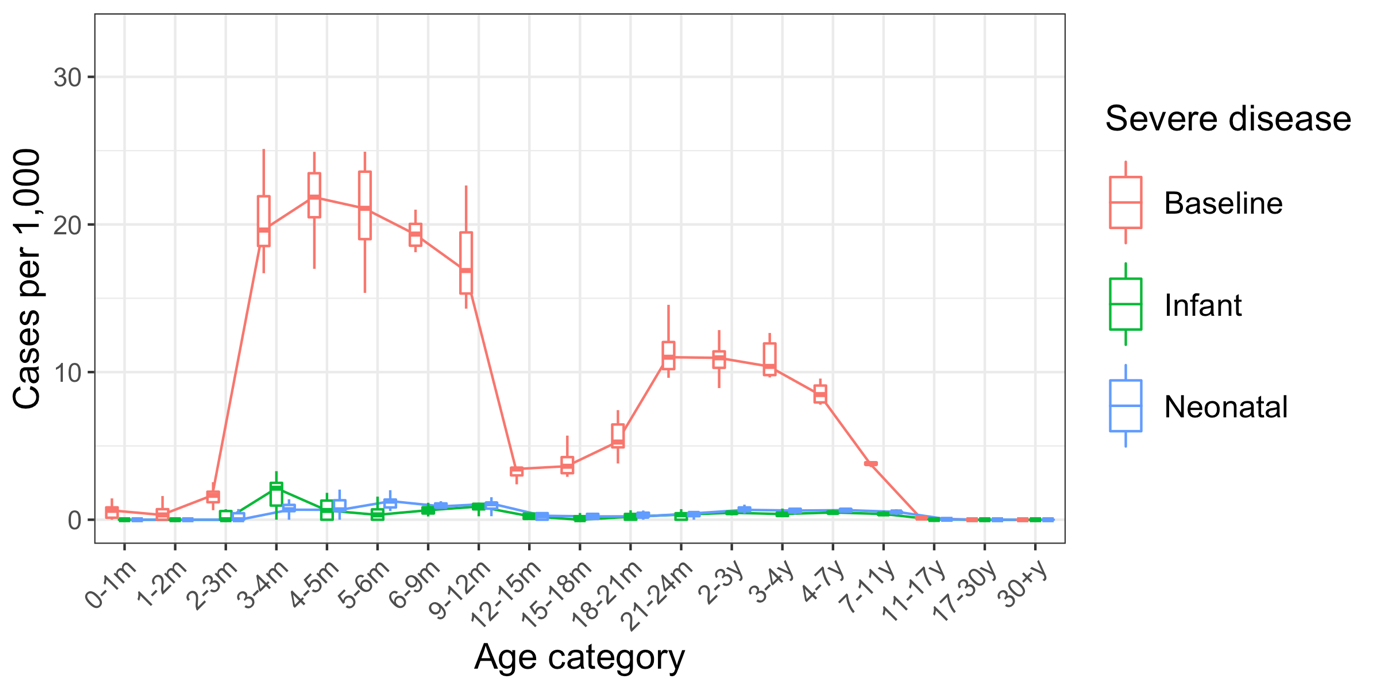


Figure S5: Annual incidence of severe rotavirus disease by age in the baseline, infant schedule and neonatal schedule scenarios. Incidence values are calculated over a five-year period, five years after the introduction of vaccination. Each boxplot shows the median, IQR and range over 10 simulation runs.

# Supplementary Table

Table S1: Demographic parameter values

| **Parameter** | **Value** |
| --- | --- |
| Daily probability of death | 0.000975000 (over 90)  0.000027625 (80 to 90)  0.000010563 (70 to 80 years) 0.000005200 (60 to 70 years) 0.000002275 (50 to 60 years) 0.000000975 (40 to 50 years) 0.000000650 (30 to 40 years) 0.000000488 (20 to 30 years)  0.000000040 (10 to 20 years)  0.000000146 (1 to 10 years)  0.000002925 (under 1 year) |
| Birth to death ratio | 2.4923 births per death |
| Marriage (age range) | 18–60 years |
| Marriage (daily probability) | 0.0001 |
| Childbearing age range | 20–58 (father)  18–48 (mother) |
| Leaving home (age range) | 18–60 years |
| Leaving home (daily probability) | 0.00001 |
